# Evaluation of Reference Genes in *Glenea cantor* (Fabricius) by Using qRT-PCR

**DOI:** 10.3390/genes12121984

**Published:** 2021-12-14

**Authors:** Ran-Ran Su, Zhong-Yan Huang, Chao-Wei Qin, Xia-Lin Zheng, Wen Lu, Xiao-Yun Wang

**Affiliations:** Guangxi Key Laboratory of Agric-Environment and Agric-Products Safety, National Demonstration Center for Experimental Plant Science Education, College of Agriculture, Guangxi University, Nanning 530004, China; srr18838933489@126.com (R.-R.S.); 15878635231@163.com (Z.-Y.H.); qincw2000@163.com (C.-W.Q.); zheng-xia-lin@163.com (X.-L.Z.); luwenlunwen@163.com (W.L.)

**Keywords:** *Glenea cantor*, qRT-PCR, reference gene, expression stability, validation

## Abstract

Kapok is the main host of *Glenea cantor* (Fabricius), which causes serious damage and is difficult to control. In severe cases, it often causes the kapok trees to die continuously, which seriously affects the results of urban landscaping. To provide reference for the functional research on related genes in *G. cantor*, we screened the stable expression of candidate reference genes at different developmental stages (i.e., eggs, larvae, pupae, and adults), in various adult tissues (i.e., head, thorax, abdomen, feet, antennae, and wings), and sexes (i.e., male pupae, female pupae, male adults, and female adults). In this study, 12 candidate reference genes (i.e., *ACTINLIKE*, *ACTININ*, *TUB*, *RPL36*, *RPL32*, *RPS20*, *TBP*, *GAPDH*, *18S rRNA*, *EF1A1*, *EF1A2*, and *UBQ*) were evaluated using different adult tissues, developmental stages, and sexes. RefFinder, geNorm, NormFinder, and BestKeeper were used to evaluate and comprehensively analyze the stability of the expression of the candidate reference genes. The results show that *RPL32* and *EF1A1* were the most suitable reference genes in the different adult tissues, and *RPL36* and *EF1A1* were best at the different developmental stages. *RPL36* and *EF1A2* were the best fit for the qRT-PCR reference genes in the different sexes, while *RPL36* and *EF1A1* were the most appropriate qRT-PCR reference genes in all samples. Results from geNorm showed that the optimal number of reference genes was two. We also surveyed the expression of *cellulase* at the different developmental stages and in the different adult tissues. Results further verified the reliability of the reference genes, and confirmed the best reference genes under the different experimental conditions. This study provides a useful tool for molecular biological studies on *G. cantor*.

## 1. Introduction

*Glenea cantor* (Fabricius) (Coleoptera: Cerambycidae: Lamiinae) is one of the dominant trunk borers of kapok trees, *Bombax ceiba* [1]. *G. cantor* adults gnaw at the bark, and larvae burrow into the trunks and branches to feed under the cortex, blocking the sap, and sometimes killing the branches or whole trees [2]. For example, in 2005, this pest killed over 10% of the trees in a *B. ceiba* plantation in Nanning, Guangxi Province, China [1]. In 2018, *B. ceiba* was discovered as a new important host plant for long-horned beetles in Nanning, Guangxi Province [3]. In severe cases, it often causes the kapok trees to die continuously, which seriously affects the results of urban greening and landscaping. *G. cantor* is widespread in China, Vietnam, Philippines, Laos, and other countries [1]. In China, the beetle is distributed in Guangxi, Guangdong, Yunnan, Hainan, and other provinces [1]. *G. cantor* lives for four generations a year, with a period of 70 days per generation in Nanning, Guangxi Province in China [4,5]. The peak period for *G. cantor* is from March to July, and the larvae mainly hibernate in the affected branches during their fourth instar [4,5]. Biological studies of *G. cantor* have mainly focused on the biological characteristics [3,4,5,6], behavior habits [2,7,8,9], prevention and control [1], artificial breeding method [9,10], and mitochondrial genome [11]. As a result, *G. cantor* is a good reproductive behavior model in the regulatory mechanism, as a polygamous long-horned beetle and R-strategist. However, basal molecular biological research remains scarce, which impedes the exploration of the molecular mechanism. Thus, transcriptome analysis was performed (unpublished data), in which the validation of the transcript expression and gene expression studies required quantitative real-time PCR (qRT-PCR) by using the validated reference genes.

The qRT-PCR is a general and prominent technique that is widely applied, and with high sensitivity and accuracy. This technique has been used to analyze the level of gene expression [12]. However, the accuracy of the qRT-PCR result is closely associated with various factors, such as the initial RNA quantity and quality, specificity of the primer, efficiency of reverse transcription, efficiency of amplification, PCR conditions, and normalization of expression [13]. Valid endogenous gene controls are often housekeeping genes, which are crucial for the accurate normalization of expression in relative qRT-PCR. However, some housekeeping genes were not stably expressed in many reported studies, which results in misleading gene expression results. Therefore, along with the wide application of sequencing techniques, extensive studies on the stability of the expression of reference genes are continuously conducted in different kinds of insects, e.g., Hymenoptera [14,15,16], Lepidoptera [17,18,19,20], Diptera [21,22,23,24], Hemiptera [25,26], and Coleoptera [27]. Recent studies in Coleoptera could provide valuable information for the selection of the candidate housekeeping gene and comparative analysis with *G. cantor* (Table 1).

In this study, 12 common candidate housekeeping genes, including *ACTINLIKE*, *ACTININ*, alpha-1-tubulin (*TUB*), ribosomal protein L36 (*RPL36*), ribosomal protein L32 (*RPL32*), 40S ribosomal protein S20 (*RPS20*), TATA-box binding protein (*TBP*), glyceraldehyde-3-phosphate dehydrogenase (*GAPDH*), 18s ribosome RNA (*18S*), elongation factor 1-alpha-1 (*EF1A1)*, elongation factor 1-alpha-2 (*EF1A2*), and ubiquitin (*UBQ*), were selected as candidate reference genes. Then, the expression levels of the genes at different developmental stages (i.e., eggs, larvae, pupae, and adults), in various adult tissues (i.e., head, thorax, abdomen, feet, antennae, and wings), and sexes (i.e., male pupae, female pupae, male adults, and female adults) of *G. cantor* were analyzed by using RefFinder (https://www.heartcure.com.au/reffinder/, 16 February 2021), geNorm, NormFinder, and BestKeeper. These analytical tools were also used to rank the stability of these candidate reference genes in the designed experimental sets. The best reference gene numbers were also suggested by geNorm. The results provided valuable essential information in the assay of the gene expression by using the relative quantitative PCR for further studies on the molecular mechanism in *G. cantor*.

## 2. Materials and Methods

### 2.1. Insect Rearing

In May 2019, the *G. cantor* larvae were originally collected from Qingxiu Mountain (22°47′ N, 108°23′ E) in Nanning City, Guangxi Province, China by cutting down damaged kapok branches. The branches were kept in cages for adult eclosion, and then the insects were reared, as introduced previously [3]. The insects were reared with ventilation under 25 ± 1 °C, 75 ± 5% relative humidity (RH), and a photoperiod of 14:10 [L:D] h.

### 2.2. Sample Collection

The candidate reference genes of *G. cantor* were evaluated using different adult tissues, developmental stages, and sexes. Three biological replicates were used in each experiment, and one replicate was introduced. Different adult tissues were sampled. A total of 40 female adults and 40 male adults were dissected for their heads (not including antennae), thoraxes, abdomens, feet, antennae, and wings. Sampling was performed at different developmental stages, namely, eggs (*n* = 200), 4th-instar larvae (*n* = 3), pupae (3 females and 3 males), and adults (3 females and 3 males). Samples of different sexes of *G. cantor* were collected, namely 3 female pupae, 3 male pupae, 3 female adults, and 3 male adults. Every sample was placed in a 1.5 mL centrifuge tube, quickly placed in liquid nitrogen, and then transferred to a −80 °C refrigerator for storage.

### 2.3. Total RNA Extraction and cDNA Synthesis

Total RNA was extracted using RNAiso Plus (TAKARA, 9109, Dalian, China) following the manufacturer’s instructions. The concentration and quality of each RNA sample were examined using a NanoPhotometer (IMPLEN, Munich, Germany). Qualified RNAs (A260/280: 1.9 to 2.1) were further synthesized into cDNA using a Prime Script RT reagent Kit with gDNA Eraser (TAKARA, RR047, Dalian, China). The cDNA of all samples was stored at −20 °C before use.

### 2.4. Primer Design

Twelve candidate reference genes from the gender-comparative transcriptome database of *G. cantor*, which were commonly used for qRT-PCR in other insect species, were evaluated. These genes included *ACTINLIKE*, *ACTININ*, *TUB*, *RPL36*, *RPL32*, *RPS20*, *TBP*, *GAPDH*, *18S*, *EF1A1*, *EF1A2*, and *UBQ* (Table 2). The qRT-PCR primers were designed by using the Primer-BLAST primer design tools on the National Center for Biotechnology Information (NCBI) website (https://www.ncbi.nlm.nih.gov/tools/primer-blast/ 16 February 2021). Then, the primers were synthesized by Sangon Biological Engineering Shanghai Synthesis (share) Co. LTD (Shanghai, China) and diluted, as introduced. The detailed primer sequences are listed in Table 2.

### 2.5. qRT-PCR

The quantitative PCR reactions were conducted on an ABI QuantStudio^TM^ 6 Flex system (Thermo Fisher Scientific, Massachusetts, USA). Each reaction was performed in a 10 µL volume with 1 µL of cDNA, 3 µL of ddH_2_O, 0.4 µL of each primer (10 µM), 0.2 µL of ROX Reference Dye II (50×), and 5 µL of TB Green Premix Ex Taq II (Tli RNaseH Plus) (2×) (TAKARA, RR820A, Dalian, China). The PCR conditions were 95 °C for 5 min, followed by 40 cycles at 95 °C for 5 s, and 60 °C for 34 s. The melting and standard curves were analyzed to ensure the efficiency and specificity of the products.

### 2.6. Analyses of Candidate Gene Expression

RefFinder (https://www.heartcure.com.au/reffinder/, 16 February 2021), geNorm, NormFinder, and BestKeeper were applied to analyze the stability of the 12 candidate reference genes. The geNorm can express the stability of the reference genes by the M value; that is, the smaller the M value is, the more stable the reference gene is. This tool can also calculate V_n/(*n*+1)_. When V_n/(*n*+1)_ < 0.15, the n reference genes in the treatment are the optimal number of reference genes [33]. NormFinder can express the stability of reference genes by the stability value (SV). The smaller the SV is, the more stable the reference gene is. This software can also obtain the optimum combination of reference genes [34]. BestKeeper can express the stability of the reference genes by standard deviation (SD), coefficient of variation (CV), and geomean (GM). When SD < 1, the gene can be used as an internal reference gene [35]. RefFinder was also used to analyze the expression of the 12 candidate genes, and includes four computational programs, geNorm [33], NormFinder [34], BestKeeper [35], and the ∆Ct method [36], to suggest a comprehensive ranking of all reference genes.

### 2.7. Expression Validation of the Reference Gene in G. cantor

The combination of the most stable (*RPL36* and *EF1A1*) and least stable (*RPS20* and *18S*) reference genes at the different developmental stages, and the combination of the most stable (*RPL32* and *EF1A1*) and the least stable (*TBP* and *18S*) genes in the different adult tissues, were validated by evaluating the expression of *cellulase*. The relative expression of the target genes was calculated using the 2^−ΔΔCt^ method. Three technical and three biological replicates were used in this analysis. Gene expression was analyzed by one-way ANOVA, followed by Tukey’s HSD test at *p* < 0.05. Data are presented as mean ± standard error of the mean (SE).

## 3. Results

### 3.1. Evaluation of Amplification Efficiency and Specificity of Primers in G. cantor

The PCR specificity of the 12 candidate reference genes was tested by analyzing the melting curves, and all genes showed a single peak. The amplification efficiency of the primers in *G. cantor* was 91.0% (*RPS20*)–109.4% (*18S*), which was within the required range of 80.0–120.0%. The regression coefficients (R^2^) of the 12 candidate reference genes ranged from 0.951 to 1.000 (Table 2). These values indicated that the selected quantitative primer pairs were well designed and had good amplification efficiency and specificity. The primers also met the requirements of fluorescence quantitative analysis, and were suitable for quantifying the candidate reference genes.

### 3.2. Expression Range of the Candidate Reference Genes in G. cantor

**Figure 1:**
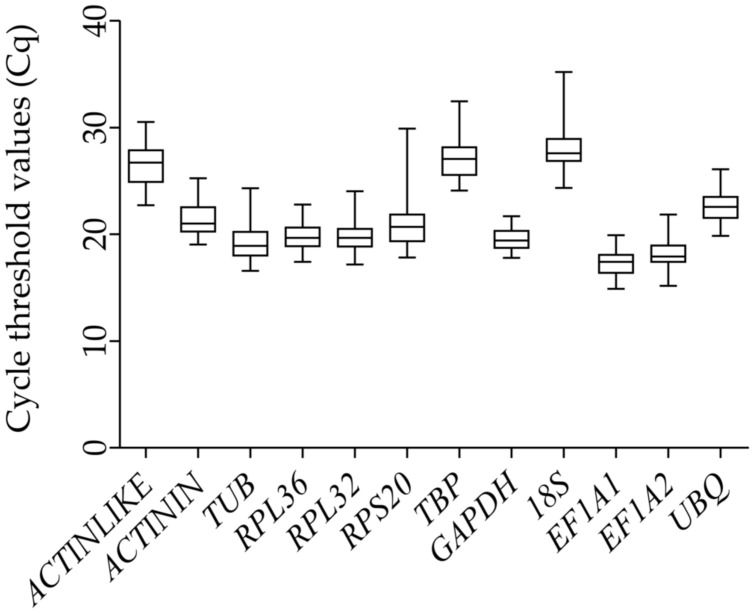
Expression levels of the candidate reference genes in the different samples of *Glenea cantor*.

The cycle threshold values (Cq) of the 12 candidate reference genes with all samples ranged from 14.891 (*EF1A1*) to 35.213 (*18S*), indicating a broad range of variation in the expression of the housekeeping genes (Figure 1). *GAPDH* had the smallest Cq range, followed by *EF1A1*, *RPL36*, and *ACTININ*. The largest Cq range was found in *RPS20*, followed by *18S*, *TBP*, and *ACTINLIKE*. Some Cq values in these four genes exceeded 30, suggesting that they might not be considered as candidate reference genes in some experimental sets.

### 3.3. Stability Analysis of the Candidate Reference Genes in G. cantor

#### 3.3.1. geNorm

GeNorm was based on the biological accuracy of relative expression levels with the comparison of Cq values. M represents a set of quantitative measurements that control the changes in gene expression levels. In addition, it is used to characterize whether the reference gene’s contribution to the stability changes of all reference genes is stable or unstable. A larger value of M shows a greater contribution to the variance of the total expression level. GeNorm can sequentially exclude the most unstable genes and retain the most stable reference gene combinations, which is an effective optimization tool. It provides a reasonable and comprehensive set of reference genes, with safer predictions for the subsequent experimental analyses of the same system [37].

**Figure 2:**
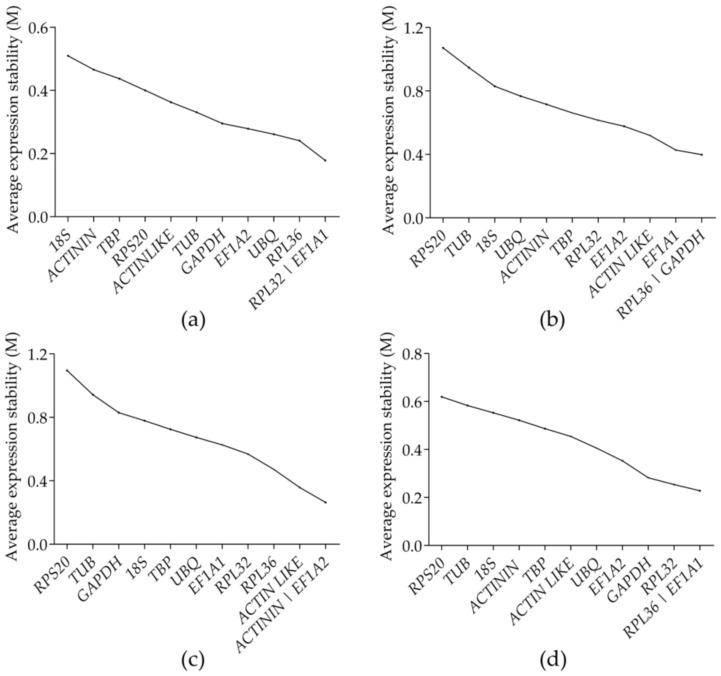
Stability of expression of the candidate reference genes in *Glenea cantor*, as calculated by geNorm: (**a**) adult tissues; (**b**) developmental stages; (**c**) sexes; and (**d**) all samples.

**Figure 3:**
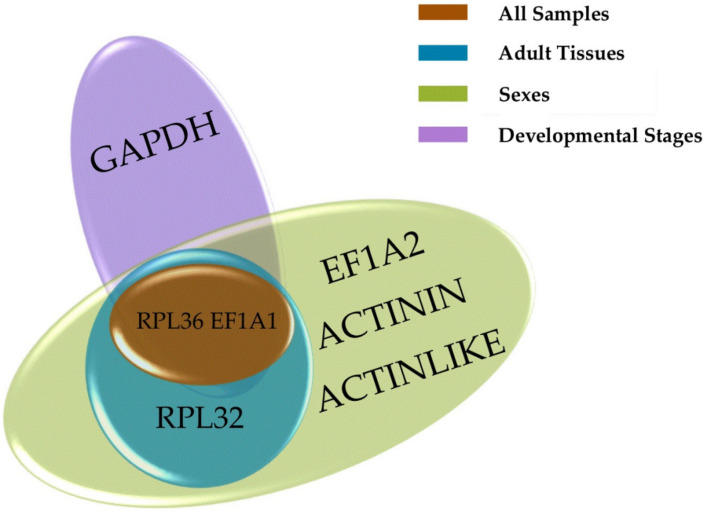
Venn diagrams showing the most stable genes identified by geNorm. The most stable genes were identified using the data under the different conditions. Each circle with a distinct color represents a different condition.

In this context, a lower M value indicates that the candidate gene is more stable. The stability (M value) of the 12 candidate reference genes was analyzed by geNorm (Figure 2). The results showed that *RPL32* and *EF1A1* were the most stable reference genes in the different adult tissues, followed by *RPL36*, and the least stable gene was *18S*. For the different developmental stages, *RPL36* and *GADPH* were the most stable reference genes, followed by *EF1A1*. *ACTININ* and *EF1A2* were the most stable reference genes in the different sexes, followed by *ACTINLIKE*. *RPL36* and *EF1A1* were the most stable reference genes in all samples, while *RPS20* was the least stable gene at the different developmental stages, in the different sexes, and in all samples. Under the three different conditions, *RPL36* and *EF1A1* may be the best choices for the reference genes (Figure 3).

**Figure 4:**
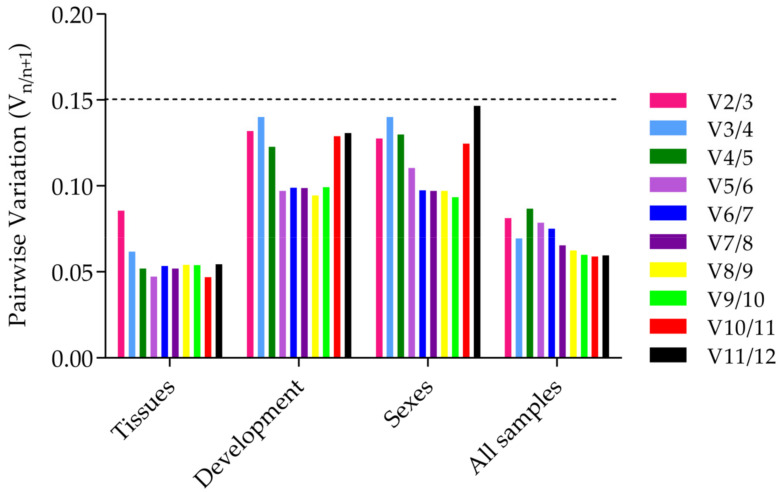
Pairwise variation (V) values using geNorm, based on the different tissues, developmental stages, sexes, and in all samples.

The value of V_n/(*n* + 1)_ was calculated using geNorm, and the number of internal reference genes needed was obtained (Figure 4). In all three treatments, V_n/(n+1)_ was less than 0.15, indicating that the optimal number of reference gene combinations was two, and a third reference gene was not needed for correction. The optimum combinations were *RPL32* and *EF1A1* in the different adult tissues, *RPL36* and *GADPH* at the different developmental stages, *ACTININ* and *EF1A2* in the different sexes, and *RPL36* and *EF1A1* in all samples.

#### 3.3.2. NormFinder

**Figure 5:**
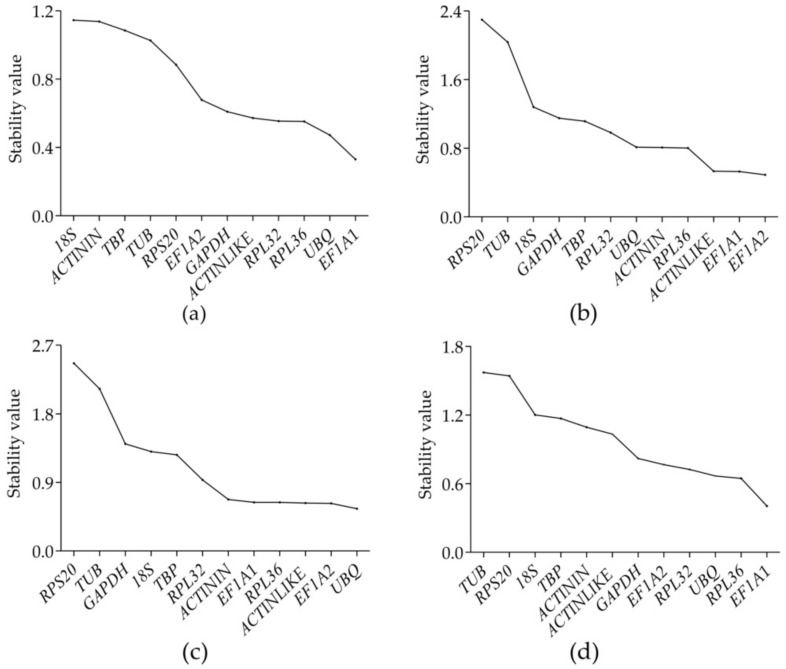
Stability of the expression of the candidate reference genes in *Glenea cantor*, as calculated by the NormFinder: (**a**) adult tissues; (**b**) developmental stages; (**c**) sexes; and (**d**) all samples.

NormFinder identifies the reference gene that exhibits the lowest expression variation by combining the within-group and between-group variation in a given sample set. The program ranks candidate reference genes according to their stability value (SV), with the lowest SV representing the highest gene expression stability [34]. The Ct values of genes were within the range of 15–35; the smaller the SV value, the more stable. NormFinder showed that *EF1A1* was the best steady reference gene in the different adult tissues, which was consistent with the results from geNorm, followed by *UBQ*, and the least stable gene was *18S*. NormFinder also showed that *EF1A2* was the best stable reference gene at the different developmental stages, followed by *EF1A1*, and *RPS20* was the wobbliest gene. *UBQ* was the most stable reference gene in the different sexes, followed by *EF1A2*, and the most volatile gene was *RPS20*. In all samples, *EF1A1* was the best stable reference gene, followed by *RPL36*, which was consistent with the results of geNorm. The results from NormFinder and geNorm indicated that *TUB* was the most unstable gene in all samples (Figure 5).

#### 3.3.3. BestKeeper

BestKeeper showed that *GAPDH* was the best stable reference gene in the different adult tissues, followed by *EF1A1*, and *TBP* was the least stable gene. *RPL36* was the best stable reference gene at the different developmental stages, which was consistent with geNorm, followed by *GAPDH. GAPDH* was the best stable reference gene in the different sexes, followed by *RPL36*. *GAPDH* was the best stable reference gene in all samples, followed by *EF1A1. TUB* was the wobbliest gene at the different developmental stages, in the different sexes, and in all samples (Table 3).

#### 3.3.4. RefFinder

**Figure 6:**
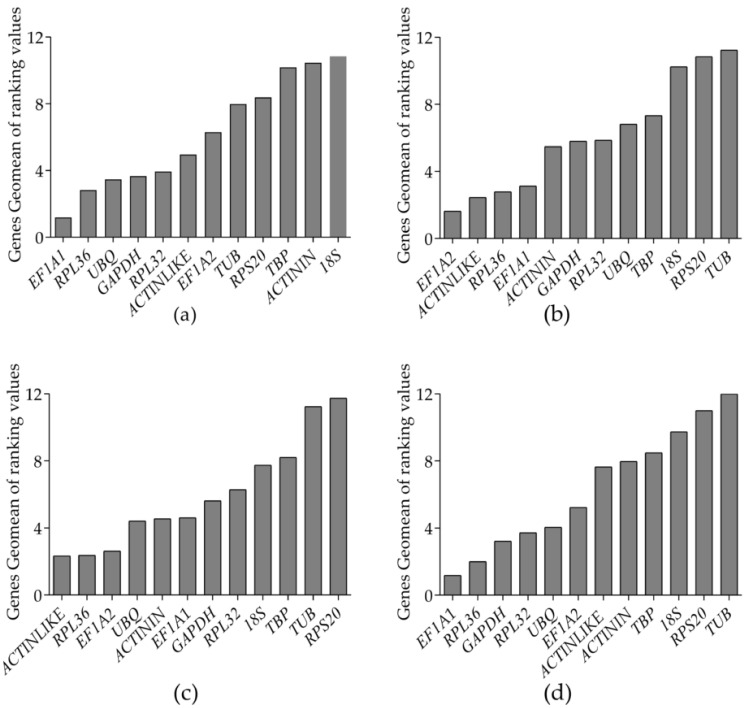
Stability of the expression of the candidate reference genes in *Glenea cantor*, as calculated by the geomean method of RefFinder: (**a**) adult tissues; (**b**) developmental stages; (**c**) sexes; and (**d**) all samples.

Comprehensive analysis using RefFinder suggested that *EF1A1* was the most stable reference gene in the different adult tissues. This result was consistent with those of geNorm and NormFinder. The next most stable reference gene in the different adult tissues was *RPL36*. At the different developmental stages, the most stable reference gene was *EF1A2*, which was in line with that of NormFinder, followed by *ACTINLIKE*. In the different sexes, the reference gene with the most stable expression was *ACTINLIKE*, followed by *RPL36*. In all samples, the most stable reference gene was *EF1A1*, which was consistent with the results from geNorm and NormFinder, followed by *RPL36* (Figure 6).

### 3.4. Validation of the Reference Gene Expression

**Figure 7:**
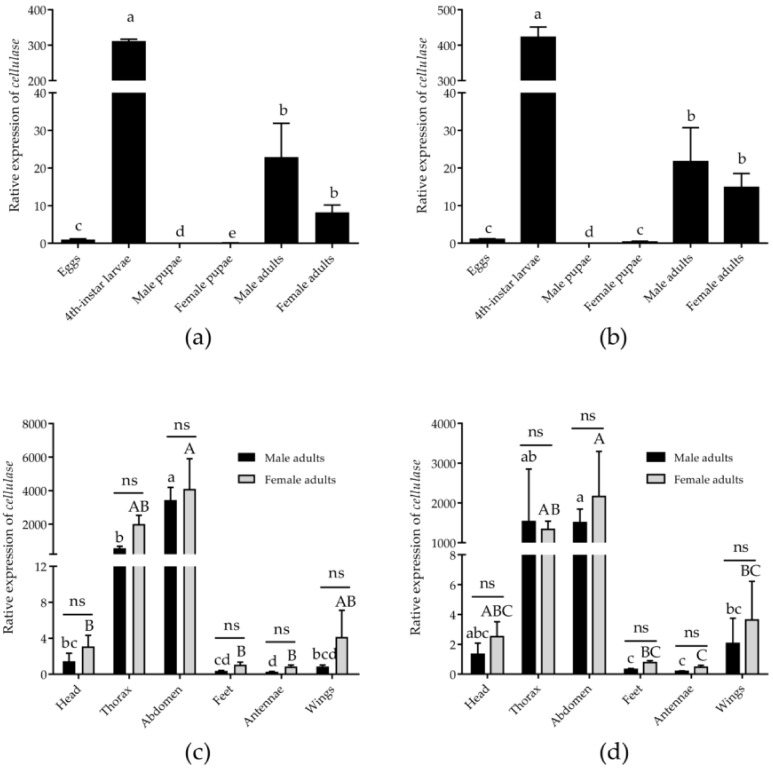
Relative expression level of the target gene (*cellulase*) in the different samples using different normalization factors (the most and least stable genes). Data are expressed as mean ± standard deviation of error. The different large (i.e., A, B, C) and lowercase (i.e., a, b, c) letters indicate that *cellulase* was significantly different in the female and male tissues (*p* < 0.05, one-way ANOVA followed by Tukey’s HSD test). Asterisks indicate the significant differences in the expression levels between the male and female adults in the same tissues, and “ns” indicates no significant differences (Independent Samples *t*-test). Normalization was performed at the (**a**) different developmental stages by using the most stable genes (*RPL36* and *EF1A1*); (**b**) different developmental stages by using the least stable genes (*RPS20* and *18S*); (**c**) different adult tissues by using the most stable genes (*RPL32* and *EF1A1*); and (**d**) different adult tissues by using the least stable genes (*TBP* and *18S*). Note: The representations with the same letters between these groups in the figure weren’t significantly different at *p* < 0.05. For example, a and b represent between the two groups significant differences at *p* < 0.05, ab and a represent between the two groups insignificant differences at *p* < 0.05.

*Cellulase* was used as a target gene to validate the applicability of the recommended reference genes. At the different developmental stages, the most stable (*RPL36* and *EF1A1*) and the least stable (*RPS20* and *18S*) reference genes were selected as the reference genes. The relative expression level of *cellulase* was significantly higher than of that in the other developmental stages in the 4th-instar larvae (*RPL36* + *EF1A1*: F_5,12_ = 121.881, *p* < 0.0001; *RPS20* + *18S*: F_5,12_ = 79.399, *p* < 0.0001) (Figure 7). By contrast, *cellulase* expression was significantly increased in the female adults when normalized with *RPS20* and *18S*. In general, *cellulase* expression was slightly lower when normalized with the optimal reference genes (*RPL36* and *EF1A1*) than with the least stable gene (*RPS20* and *18S*) (Figure 7). In the different female adult tissues, the most stable recommended reference gene combinations (*RPL32* and *EF1A1*) and the most unstable genes (*TBP* and *18S*) were the reference genes. The relative expression level of *cellulase* in the abdomen was higher than those in the other tissues (*RPL32* + *EF1A1*, H_5,12_ = 12.883, *p* = 0.024; *TBP* + *18S*, H_5,12_ = 13.585, *p* = 0.018) (Figure 7). However, in the male adult tissues, the *cellulase* expression increased significantly when the data were normalized with the most unstable genes (*TBP* and *18S*) in the thorax and wings (Figure 7). These results showed that the best reference genes (*RPL32* and *EF1A1*) were more reliable than the least stable genes (*TBP* and *18S*) for normalizing the expression of *cellulase* (Figure 7). The results showed that selecting reference genes with stable expression could increase the reliability and stability of the experimental results.

## 4. Discussion

The screening of the stable expression of candidate reference genes in insects has been extensively reported. This research is necessary for studies on molecular biology, especially for basic gene expression. Several studies have focused on the screening of the internal reference gene for Coleoptera by using qRT-PCR (Table 1). However, the number of Coleoptera species with solid reference genes is far from sufficient, considering the total number and importance of these species. We have focused on *G. cantor* and realized remarkable progress in our studies on its biology and reproduction. Thus, we would like to further study this species on the molecular level. We established a gender-comparative transcriptome database for *G. cantor*, from which 12 candidate reference genes were selected for further evaluation.

The qRT-PCR is one of the most sensitive and widely used methods for gene quantification. This process can have high sensitivity, high sequence specificity, real-time detection, fast analysis, and accurate determination of detected substances in the samples [38,39,40]. The qRT-PCR is often used for genetic authentication and screening of the internal reference gene [41,42]. The variation in the expression of these 12 reference genes in *G. cantor* is new supplementary evidence that proves the necessity to evaluate the stability of the reference gene, rather than to use generally acknowledged housekeeping genes.

Housekeeping genes are necessary for the maintenance of basic cell functions and for cell survival. These genes can be expressed in all cells of an organism under normal conditions, regardless of tissue type, stage of development, cell cycle status, or external signals [43]. The stable housekeeping genes should be closely related to the insect species and sampling conditions, according to our comparative results with other studies. In this study, *RPL32* and *EF1A1* showed the most stable expression as qRT-PCR reference genes in the different adult tissues of *G. cantor.* This result was contrary to those of *Coccinella septempunctata*, *Henosepilachna vigintioctomaculata*, and *Hippodamia convergens. Elongation factor 1-alpha* had one of the most stable expressions in *Propylea japonica* and *A. leii* [44,45,46,47,48]. At the different developmental stages, the most stable expression gene was *EF1A* in *C. septempunctata*, *H. convergens*, and *P. japonica* [45,47,48]. In *G. cantor*, *EF1A1* was also one of the most suitable qRT-PCR reference genes. Thus, this gene may be stable in the developmental stage expression in Coleoptera, and suitable for treatment during the developmental stages. *RPL36* and *EF1A2* were the most suitable qRT-PCR reference genes in the different sexes of *G. cantor*, but this result was not consistent with those of *Harmonia axyridis* [49]. *Elongation factor 1-alpha* was one of the most stable expressions in *A. leii* and *P. japonica* [27,45].

The number of reference genes used for accurate standardization is also a focal point in evaluating the stability. From the geNorm results, the V_n/(n+1)_ value was less than 0.15 in all treatments, and the optimal number of reference gene combinations was calculated to be two. In insects, the numbers of reference genes used to verify the expression of target genes were one, two, three, or more. At least one internal reference gene should be used to normalize the expression levels, but the calibration and standardization of qRT-PCR data by a single internal reference gene may affect the accuracy of the results. The reference gene of *Conogethes pinicolalis* was one during the larval development stages (*GAPDH* or *ribosomal protein 49* gene (*RP49*)) and adult tissues (*RP49* or *ribosomal protein L13* gene (*RPL13*)) [50]. The use of two internal reference genes is optimal and widely used. For example, the reference genes of *Tuta absoluta* Meyrick was two (developmental stages: *GAPDH* and *RPS11*; different tissues, *RPL28* and *RPL10*; 20E treatment: *EF1α* and *RPL28*; insecticide treatments: *EF1α* and *RPL7A*) [51]. The number of optimal combinations of the reference genes in *Trichogramma chilonis* was also two, with optimal combinations of *RPS23* and *EF2* for the developmental stages, *ZFP268* and *EF2* for feeding with different diets, *ZFP268* and *RPL13* for temperature treatments, and *EF2* and *RPL44* for the insecticide treatments [52]. Three or more internal reference genes may increase the experimental workload and may waste reagents. However, multiple internal reference genes have also been used. For example, the number of developmental stage-specific reference genes was three for *T. dendrolimi* Matsumura (*SOD*, *GAPDH*, and *ACTIN*) [53]. The number of reference genes was five in *Apis mellifera* under different viral infections (IAPV infection: *ache2*, *rps18*, *β-actin*, *tbp*, and *tif*; CSBV treatment: *rpl14*, *tif*, *rpsa*, *ubc*, and *ache2*; and dsRNA treatment: *Rpl14*, *tif*, *rps18*, *ubc*, and *α-tubulin*) [54]. In conclusion, the principle for the internal reference genes is that the ratio of the expression levels of the two optimal internal reference genes is consistent, regardless of the experimental conditions, and was not affected by differences in the gene expression [33]. In this study, under all treatments, the optimal number of reference gene combinations was two.

Various factors could influence the accuracy of the normalization of the target gene. Although the stable reference gene has been recommended in this research, we suggest additional verification work, rather than the direct use of our mentioned gene pairs. For instance, several stable reference genes have been reported in the southern green stink bug *Nezara viridula* by different researchers previously (*40S ribosomal protein S17*), while in the gene expression studies of RNA interference (RNAi), *60S ribosomal protein L12* was added as the most stable gene [55]. For *A. leii*, the most suitable reference genes were *S27Ae* and *Reep5* at the different life stages, and *Ef1a* and *Odc1* in the different tissues. However, in the RNAi studies, *Tub* and *Odc1* were the most suitable reference genes [44]. Some of the internal reference genes screened by us can be directly used in RNAi experiments after verification. For the tissues, the reference genes used by *Thermobia domestica* were *RPS26* and *RPL32* in the different tissues under dsRNA microinjection treatment [56]. RNAi studies of specific target genes usually also screen the suitable reference genes. *Dendroctonus frontalis* has been shown to be stable in the RNAi experiments with *rps18* and *ef1a* [57]. In the experiment, the selection of internal reference genes was the basis for the subsequent study of genes related to *G. cantor* by using the RNAi technology and other factors to reduce the accuracy of the normalization of target genes. *Cellulase*, which belongs to glycoside hydrolysis, is crucial to wood digestion and nutrition in *G. cantor*, similar to other wood-digesting longhorn beetles [58]. *Cellulase* is one of the primary RNAi target genes from the point of view of genetic pest control, and needs further exploration.

## 5. Conclusions

Twelve candidate reference genes in *G. cantor* were selected to evaluate their stability under three experimental conditions. The analytical results from geNorm showed that the optimal number of reference genes was two. This result was consistent with the popular number of reference genes for the accurate normalization of target genes. However, different experimental conditions with four analytical methods would provide slightly varied ranking orders. In the different adult tissues, *RPL32* and *EF1A1* were the most suitable reference genes for qRT-PCR. At the different developmental stages, *RPL36* and *EF1A1* were the most suitable ones, while *RPL36* and *EF1A2* were the most suitable ones in the different sexes. All samples showed that *RPL36* and *EF1A1* were the most suitable reference genes. We also suggest a validation of these recommended reference genes, rather than their direct application in experiments different from our experimental sets.

## Figures and Tables

**Table 1:** Suggested stable reference genes in some Coleoptera species with different experimental sets.

Species	Condition	Optimal Reference Gene	Reference
*Anthonomus eugenii* Cano	developmental stages	*EF1-α*, *18S* and *RPL12*	[28]
	sexes	*RPS23* and *RPL12*	
	low temperature	*GAPDH* and *α-TUB*	
	high temperature	*α-TUB* and *RPS23*	
	all temperatures	*α-TUB* and *GAPDH*	
	starvation	*RPL12* and *α-TUB*	
*Aquatica leii*	tissues	*α-tubulin* and *β-tubulin*	[27]
	temperatures	*β-tubulin*, *EF1A* and *GST*	
	sexes	*β-actin* and *EF1A*	
	developmental stages	*β-tubulin GST* and *GAPDH* and *SDHA*	
	larvae exposed to different concentrations of benzo(a)pyrene	*α-tubulin* and *EF1A*	
*Sympiezomias velatus*	tissues	*TUB*, *TUA*, *RPS20* and *RPL12*	[29]
*Monochamus alternatus*	different chemosensory tissues at different developmental stages and in different genders	*GAPDH* and *TUB*	[30]
*Melanotus cribricollis*	infectious conditions of *Metarhizium pingshaense*	*PRS27* and *RPS3*	[31]
*Tribolium castaaneum* (Herbst)	developmental stages	*RPS6*, *RPL13a*, *RPS3* and *RPS18*	[32]

**Table 2:** Primers of *G. cantor* used for qRT-PCR analysis.

Gene Name (Abbreviation)	GenBank Accession Number	Primer Sequence (5′–3′)	Amplicon Size (bp)	PCR Efficiency	Regression Coefficient (R^2^)
*ACTINLIKE*	MW462107	F: TTCAAACTGGCGGAAGGGTT R: GGGCCGGTCTTATATCCACG	100	1.005	0.996
*ACTININ*	MW462108	F: GTCAACGCAAGACTGCTCCA R: TCAGGGCGATGACGATGAAT	106	0.992	0.998
*TUB*	MW462097	F: CTACACCATCGGCAAGGAAA R: CTCCGAAAGAGTGGAAGATCAG	107	0.947	0.997
*RPL36*	MW462098	F: GAAATTCGTGCGAGACCTCATC R: GGCGGCGCTTAAGGAATTTA	119	0.959	0.998
*RPL32*	MW462099	F: TCAAGGGCCAGTTCTTGATG R: ACTTTCCTGAATCCGGTTGG	85	0.960	0.999
*RPS20*	MW462100	F: CCAGTACGTATGCCCACAAA R: ACCTGTCCCAGGTCTTAGAA	82	0.910	0.999
*TBP*	MW462101	F: GAGACTGGTGCTGCTCATATT R: GCGCATCTTTGATGTCTTGTC	80	0.927	0.984
*GAPDH*	MW462102	F: CAAGGCTGGAATCTCTCTCAA R: GTTGATCAAGTCGATGACCCT	97	0.938	0.995
*18S*	MW462103	F: CGGTGGAAAGAGAGGTAGAAG R: CAACGCCGAAATGCTGATAG	104	1.094	0.961
*EF1A1*	MW462104	F: TGGCGATGCTGCCATTAT R: GGACAGCGAAACGTCCTAAT	92	1.081	0.951
*EF1A2*	MW462105	F: GGAGAATTCGAAGCTGGTATCT R: CGCCAACAATGAGTTGTCTTAC	92	0.981	0.998
*UBQ*	MW462106	F: AGGTGGCATGCAGATCTTT R: CTTGGCCTTGACGTTCTCTAT	94	1.011	1.000
*cellulase*	OL757647	F: GCGCTTGGGCTGAAAATTTG R: ACTACACTGGGCTGCTGATTAC	148	0.966	0.998

Note: F, forward primer; R, reverse primer; PCR efficiency and regression coefficient (R^2^), as calculated from the standard curve.

**Table 3:** Stability of gene expression in *Glenea cantor*, as ranked by BestKeeper.

	Tissues			Developmental Stages			Sexes			All Samples		
Rank	Gene Name	SD (CP)	CV (CP, %)	Gene	SD (CP)	CV (CP, %)	Gene	SD (CP)	CV (CP, %)	Gene Name	SD (CP)	CV (CP, %)
1	*GAPDH*	0.87	4.47	*RPL36*	0.76	3.88	*GAPDH*	0.67	3.45	*GAPDH*	0.83	4.26
2	*EF1A1*	0.92	5.33	*GAPDH*	0.76	3.90	*RPL36*	1.02	5.21	*EF1A1*	0.97	5.66
3	*RPL32*	0.98	4.96	*RPL32*	1.01	5.20	*EF1A1*	1.14	6.74	*RPL32*	1.02	5.20
4	*EF1A2*	0.99	5.56	*EF1A1*	1.03	6.02	*RPL32*	1.30	6.64	*RPL36*	1.05	5.29
5	*TUB*	1.01	5.30	*TBP*	1.25	4.72	*ACTINLIKE*	1.33	5.28	*EF1A2*	1.16	6.38
6	*UBQ*	1.07	4.77	*ACTINLIKE*	1.28	5.04	*ACTININ*	1.47	6.84	*UBQ*	1.31	5.79
7	*RPL36*	1.15	5.81	*EF1A2*	1.44	7.68	*TBP*	1.52	5.71	*ACTININ*	1.42	6.61
8	*18S*	1.24	4.43	*RPS20*	1.51	7.39	*EF1A2*	1.55	8.34	*TBP*	1.48	5.45
9	*ACTININ*	1.28	6.03	*ACTININ*	1.68	7.68	*UBQ*	1.84	8.19	*18S*	1.49	5.32
10	*ACTINLIKE*	1.30	4.81	*UBQ*	1.85	8.05	*18S*	1.89	6.80	*ACTINLIKE*	1.51	5.70
11	*RPS20*	1.40	6.63	*18S*	2.05	7.28	*RPS20*	1.94	9.35	*RPS20*	1.51	7.24
12	*TBP*	1.51	5.53	*TUB*	2.46	12.17	*TUB*	2.52	12.89	*TUB*	1.56	8.05

Note: SD, standard deviation; CV, coefficient of variation; CP, Mallows’s Cp.

## Data Availability

The data presented in this study are available on request from the corresponding author.
